# Sample Copies

**Published:** 1878-03-20

**Authors:** 


					﻿EDITORIAL AND MISCELLANEOUS.
SAMPLE COPIES.
The medical gentlemen to whom the present
number of this journal is sent as a sample, are re-
quested to subscribe. Try “The Record.”
Send on your names at once, and get the numbers
from January that your volume may be complete.
We guarantee that you will find an ample equiva-
lent for the small price of subscription.
				

